# Using Machine Learning Models to Predict Hydroponically Grown Lettuce Yield

**DOI:** 10.3389/fpls.2022.706042

**Published:** 2022-03-03

**Authors:** Ali Mokhtar, Wessam El-Ssawy, Hongming He, Nadhir Al-Anasari, Saad Sh. Sammen, Yeboah Gyasi-Agyei, Mohamed Abuarab

**Affiliations:** ^1^Department of Agricultural Engineering, Faculty of Agriculture, Cairo University, Giza, Egypt; ^2^State Key Laboratory of Soil Erosion and Dry Land Farming on Loess Plateau, Institute of Soil and Water Conservation, Chinese Academy of Sciences and Ministry of Water Resources at Northwest University of Agriculture and Forestry, Xianyang, China; ^3^School of Geographic Sciences, East China Normal University, Shanghai, China; ^4^Irrigation and Drainage Department, Agricultural Engineering Research Institute, Agricultural Research Center, Giza, Egypt; ^5^Department of Civil Engineering, Environmental and Natural Resources Engineering, Lulea University of Technology, Lulea, Sweden; ^6^Department of Civil Engineering, College of Engineering, University of Diyala, Baquba, Iraq; ^7^School of Engineering and Built Environment, Griffith University, Nathan, QLD, Australia

**Keywords:** machine learning, deep learning, DNN, yield prediction, food safety 2

## Abstract

Prediction of crop yield is an essential task for maximizing the global food supply, particularly in developing countries. This study investigated lettuce yield (fresh weight) prediction using four machine learning (ML) models, namely, support vector regressor (SVR), extreme gradient boosting (XGB), random forest (RF), and deep neural network (DNN). It was cultivated in three hydroponics systems (i.e., suspended nutrient film technique system, pyramidal aeroponic system, and tower aeroponic system), which interacted with three different magnetic unit strengths under a controlled greenhouse environment during the growing season in 2018 and 2019. Three scenarios consisting of the combinations of input variables (i.e., leaf number, water consumption, dry weight, stem length, and stem diameter) were assessed. The XGB model with scenario 3 (all input variables) yielded the lowest root mean square error (RMSE) of 8.88 g followed by SVR with the same scenario that achieved 9.55 g, and the highest result was by RF with scenario 1 (i.e., leaf number and water consumption) that achieved 12.89 g. All model scenarios having Scatter Index (SI) (i.e., RMSE divided by the average values of the observed yield) values less than 0.1 were classified as excellent in predicting fresh lettuce yield. Based on all of the performance statistics, the two best models were SVR with scenario 3 and DNN with scenario 2 (i.e., leaf number, water consumption, and dry weight). However, DNN with scenario 2 requiring less input variables is preferred. The potential of the DNN model to predict fresh lettuce yield is promising, and it can be applied on a large scale as a rapid tool for decision-makers to manage crop yield.

## Introduction

The changing conditions of climate and weather patterns during the past years have fueled the current problems of land and water scarcity and continue to cause harm in the agricultural sector (Majid et al., 2021). Globally, the agricultural sector is the largest consumer of water comprising about 70% of the total demand, but 70% of this is returned as wastewater through the different processes (Kloas et al., 2015; Murad et al., 2017). While *per capita* drinking water is about 2–5 L/day, it requires about 5,000 L of water to produce daily dietary needs per person (Manju et al., 2017). The development of sustainable plans has become a global focus, and a circular economy is the order of the day (Wei et al., 2019).

Without a doubt, the use of modern technologies has increased ability of mankind to meet the latest challenges of limited resources. Hydroponic systems are considered as an alternative to traditional agricultural systems (Majid et al., 2021). Safety, sustainability, and policy issues associated with water and agriculture are fundamental to Egyptian interests. Irrigated agriculture is the main user of water resources in most parts of the world. Stress on water availability and associated impacts among competing user groups in the region are increasing due to population growth, development, environmental, and wildlife concerns (Abd-Rbo et al., 2015). Therefore, the application of modern agricultural techniques of hydroponic and aeroponics without the need for soil is on the increase (Mehra et al., 2018). Hydroponic systems can increase water productivity and maintain the quality of production. Therefore, they should be implemented on any scale to support the environment and agriculture (El-Ssawy et al., 2020). Artificial intelligence (AI), such as neural networks, has been applied in hydrology to deal with complex phenomena (Elbeltagi et al., 2020; Abdel-Fattah and Abdo, 2020; Mokhtar et al., 2021) and is also used to control the growth of hydroponic plants (Mehra et al., 2018). For some systems, such as the nutrient film technique (NFT), a fresh solution of nutrients is continuously supplied to the crops to compensate for the uptake of nutrients and water by the plants. In some systems, the input of nutrients is based on the nutrient/water uptake ratio concept, i.e., nutrient weight per unit volume of water absorbed (Sonneveld and Voogt, 2001; Neocleous and Savvas, 2019).

Lettuce grows much faster in aeroponics compared to a floating system, probably due to the higher dissolved oxygen level in the nutrient solution (Puccinelli et al., 2021). Hydroponic systems can be automated using Internet of Things technology, and machine learning (ML), a subset of AI, is very beneficial in this regard. However, the use of ML in hydroponic/aeroponic systems to automate plant growth has received less research (Araújo et al., 2019). Recently, there have been many approaches to estimate crop yield based on conventional methods, including models of process-oriented crop simulation and statistical-based models analyzing crop production and explanatory variables (Johnson, 2014; Cai et al., 2019). Conventional statistical-based methods or specific response functions linking yield and independent variables provide an alternative to forecast yield due to their simpler computation and higher interpretation power (Qader et al., 2018). However, there are some problems with conventional empirical prediction models because they tend to be applicable to local conditions and the generalization for other areas is limited (Qader et al., 2018; Folberth et al., 2019). ML is a “black-box” with complicated functions but has the capability for dealing with complex relationships between the independent and the dependent variables (Kamir et al., 2020; Cao et al., 2021). In recent years, ML techniques have been used in agricultural research fields, such as classification of crop and monitoring of growth and prediction of yield in some countries (Sadeghipour et al., 2013; Shah et al., 2019; Wolanin et al., 2019). The ground is now set for future sustainable agriculture that is data-driven to feed AI and robots (Saiz-Rubio and Rovira-Más, 2020).

The ML is improving the ability of computers to perform actions on their own after they have been trained for a specific task. For machines to think like humans, they should first learn like human beings. The mind of a human being makes decisions based on past experiences, i.e., the data of the past that one has been exposed to. ML algorithms have different uses in hydroponics, such as to control plant growth, electrical conductivity (EC) values, and the constituents of the nutrient solution (Mehra et al., 2018). It instructs computers to perform complicated tasks through regression, diagnosis, planning, and recognition by learning from historical data. Thus, data and algorithms are considered fundamental to performance of ML models. Higher quality data and larger data sizes are instrumental for the accuracy of ML models. It is also necessary to apply suitable algorithms to achieve solutions to different problems containing different types of datasets (Kang et al., 2020). For example, Johnson (2014) applied a regression tree (RT) for predicting yields of soybean and maize at the county-level in the United States. In Australia, Cai et al. (2019) compared the three improved ML models [i.e., support vector machine (SVM), random forest (RF), and neural network (NN)] and the method of traditional regression [i.e., Least Absolute Shrinkage and Selection Operator (LASSO)] for the prediction of wheat yield. Their results showed that ML methods were better than the traditional regression method.

Jeong et al. (2016) predicted the yield of wheat, maize, and potato by applying RF and multiple linear regression (MLR). They concluded that RF was better than MLR in predicting crop yields. Fukuda et al. (2013) also applied RF to predict yields of mango fruit with a successful outcome. Deep learning (DL), a subset of NN, has multiple layers and progressively extracts higher-level features from the raw input data (Lecun et al., 2015; Khaki and Wang, 2019). You et al. (2017) used convolutional neural networks (CNNs) and recurrent neural networks (RNNs) to predict soybean yield based on a sequence of remotely sensed images. Furthermore, a deep neural network (DNN) was applied to predict maize yield during 2008–2016, and the results showed that DNN was clearly better than LASSO, shallow neural network (SNN), and RT (Khaki and Wang, 2019). Kim et al. (2019) applied a DNN model to predict corn and soybean yield during 2006–2015. In Argentina, Khaki and Wang (2019) developed a DNN for predicting soybean yields.

The initial cost for establishing a hydroponic system is very high, making it imperative to predict crop yield before establishment using models, such as ML. Therefore, the objectives of this study were to (1) apply four ML models to predict fresh head weight (yield) of lettuce under controlled greenhouse conditions subject to three input scenarios consisting of the combinations of input variables and (2) identify the best model scenarios.

## Materials and Methods

### Experimental Treatments

**FIGURE 1:**
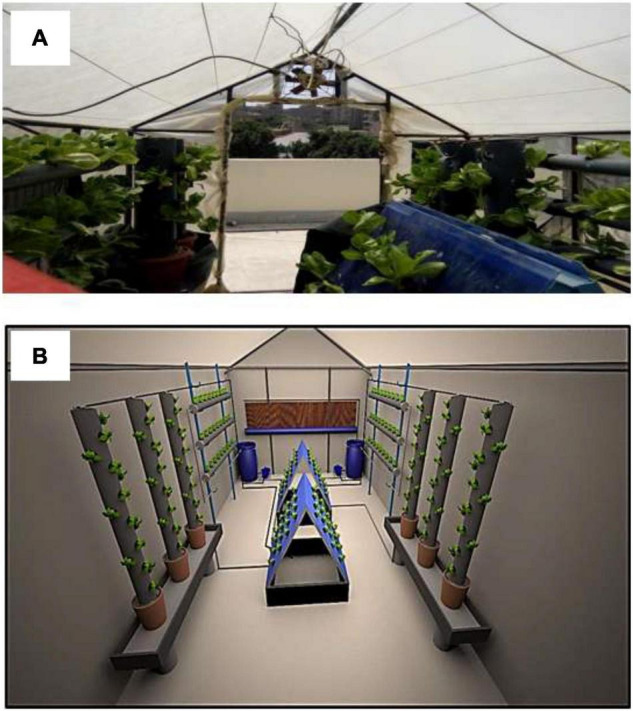
Components of the experimental setup. **(A)** Photograph. **(B)** Computer graphics.

**FIGURE 2:**
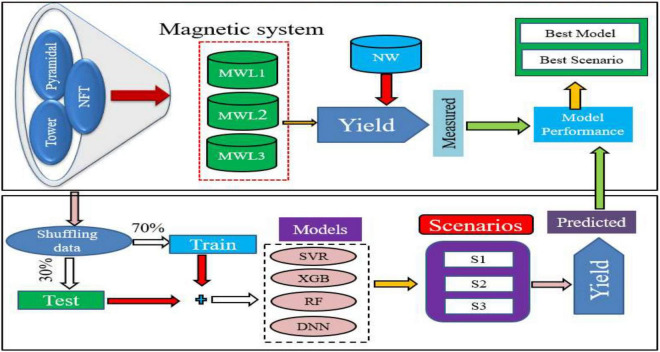
Flowchart of the treatments implemented and models applied.

The experiment was conducted in a controlled greenhouse (2.0 m wide, 3.5 m long, and 2.5 m height) environment made with an iron frame covered with a polyethylene sheet at the Agricultural Engineering Research Institute, Agricultural Research Center, Giza, Egypt, during the growing season in 2018 and 2019. It contained three hydroponics systems (i.e., suspended NFT system, pyramidal aeroponic system, and tower aeroponic system) as shown in Figure 1, subjected to three different magnetic levels (MWL1 = 3,800 gauss, MWL2 = 5,250 gauss, and MWL3 = 6,300 gauss) (Figure 2). The nutrient solution was pumped from an irrigation storage tank through 16-mm polyethylene pipes connected to each system by a 1-hp pump, and the irrigation rate was 10 L/day for 6 h.

The suspended NFT system consisted of 150-cm-high vertical iron stands that support three horizontal pipes each of 250 cm length and 10.16 cm diameter. Each pipe had holes with 5 cm diameter at 20-cm intervals containing the hydroponic cups that housed the plants. The pyramidal aeroponic system consisted of 1 m^2^ iron frames, two put together to make a V-shaped structure and placed on an iron tank (1 m wide, 1 m long, and 0.5 cm deep). The iron frames were covered with high-density plastic sheets on both sides forming a triangular pyramid, the plants being housed in the plastic sheet. A gutter at the bottom of the pyramid collected the nutrient solution which was then redirected to the irrigation storage tank. Four foggers of 0.5 m diameter, discharging at 6 L/h under 2 bar pressure, were installed inside the system. The tower aeroponic system was made of pipes of 15.24 cm diameter and 1.5 m height. Also with this system, the plants were placed at 20 cm intervals in hydroponic cups within holes of 5 cm diameter. The nutrient solution was pumped from a tank to the foggers installed above the system through a polyethylene pipe of 16 mm diameter. The same type of foggers was used for both the pyramidal and the tower aeroponic systems.

The lettuce (cv. *LimorHyb*.) plants were obtained from the Institute of Horticulture Research, Giza, Egypt. In the hydroponic systems, the plants were grown in high-density sponges of 3 cm thick. They were cultivated for 3 weeks in 5 cm deep cups filled with nutrient solution to generate complete rooting. The plants were placed in different hydroponics systems after rooting on April 01, 2018, and March 01, 2019. Irrigation water was sourced from two tanks filled with a nutrient solution in the environmentally controlled greenhouse. The EC of the nutrient solution was approximately 1.5 dS/m which also had the following chemical properties: *N* = 51, *P* = 219.29, *K* = 358.3, Ca = 135, Mg = 45, Fe = 2.7, Mn = 0.75, Cu = 0.375, Zn = 0.113, *B* = 0.188, and Mo = 0.009 (Jackson and McGonigle, 2005).

### Climate Conditions

The range of temperature during the two seasons was 23–25 and 20–22°C, and the relative humidity was 60–65%. These weather conditions were controlled and monitored by the greenhouse tools (i.e., cooling pad, suction van, and monitoring sensor) and were checked by a Hygrometer Thermo-Anemometer Model 407412 (accuracy ±0.8°C and ±3%) and monitoring sensor CSP60BA252M with a nominal resistance of 2,500 ohms. Light intensity was 1981:1992 in the lux unit, and it was measured by light meter Model YK-10LX (accuracy ±5% and 4 days).

### Plant Variables and Scenarios

The systems were designed to contain 64 plants per square meter in each system. The harvest occurred after 50 days from planting in the systems at the same time. For each harvest, three plants were taken from each system. Then, the explanatory features, or variables used interchangeably, of leaf number, stem length, stem diameter, and dry weight, as well as the water consumption, and the dependent feature of fresh head weight (yield) were recorded. Descriptive statistical analysis of the collected data during the growing season of 2 years is shown in Table 1 for the three complete datasets. The explanatory features were divided into three scenarios: scenario 1 (leaf number and water consumption), scenario 2 (leaf number, water consumption, and dry weight), and scenario 3 (leaf number, water consumption, dry weight, stem length, and stem diameter, i.e., all input variables) (Table 2).

**TABLE 1:** Descriptive statistical analysis of the collected data.

	Mean	Max	Min	SD	Q1	Q3
Stem diameter	22.05	28.20	17.00	2.84	19.98	23.98
Leaf number	26.88	37.00	21.00	3.51	24.00	29.00
Stem length	41.15	52.00	32.00	4.28	38.00	43.00
Dry weight	18.20	27.90	13.10	3.17	16.25	19.05
water/area	0.32	0.42	0.25	0.05	0.26	0.34
Fresh head weight	329.81	416.20	275.20	36.48	301.73	346.10

**TABLE 2:** Summary of the combination of the input variables for the applied models.

Scenario	Model				Input variables combination
1	SVR1	XGB1	RF1	DNN1	Leaf number, water consumption
2	SVR2	XGB2	RF2	DNN2	Leaf number, water consumption, dry weight
3	SVR3	XGB3	RF3	DNN3	Leaf number, water consumption, dry weight, stem length, stem diameter

*SVR1, XGB1, RF1, and DNN1 for the first scenario, 2 is the second scenario, and 3 is the third scenario.*

### Machine Learning Models

#### Support Vector Machine

The SVM is a supervised learning algorithm that can also be used as a regression model. The main objective is to minimize the errors and individualize the hyperplane that increases the tolerance limit. The approximated function in the algorithm of SVM is given as follows:


(1) $$f\left(x\right)=\omega \varphi \left(x\right)+b$$

where φ (x) is a feature space of higher dimension converted from the input vector *x*, ω represents the weights vector, and *b* are thresholds that are estimated by minimizing the following regularized risk function:


(2) $$R\left(C\right)=C\frac{1}{n}\sum_{i=1}^{n}L\left(d_{i},y_{i}\right)+\frac{1}{2}{∥\omega ∥}^{2}$$

where *C* is the penalty parameter of the error, *d*_*i*_ is the desired value, *n* is the number of observations, and $C\frac{1}{n}\sum_{i=1}^{n}L\left(d_{i},y_{i}\right)$ is the empirical error in which the function *L*_ε_ is determined as follows:


(3) $$L_{\varepsilon }\left(d,y\right)=\left|d-y\right|-\varepsilon \left|d-y\right|\geq \varepsilon \;\mathrm{or}\;0\;\mathrm{otherwise}$$

where $\frac{1}{2}{∥\omega ∥}^{2}$ is the so-called regularization term and ε is the tube size. The approximated function of Equation (1) is expressed in an explicit form by introducing Lagrange multipliers and exploiting the optimality constraints as follows:


(4) $$f\left(x,\alpha_{i},\alpha_{i}^{*}\right)=\sum_{i=1}^{n}\left(\alpha_{i}-\alpha_{i}^{*}\right)k\left(x,x_{i}\right)+b$$

where *k*(*x*, *x*_*i*_) is the kernel function. Vapnik (2016) and Fan et al. (2018) have provided detailed information and the computational procedures of the SVM algorithm.

#### Extreme Gradient Boosting

The extreme gradient boosting (XGB) algorithm proposed by Chen and Guestrin (2016) is a novel implementation method for Gradient Boosting Machine which is based on RTs. The algorithm depends on the “boosting” idea which combines all the predictions of a set of “weak” learners to develop a “strong” learner during strategies of additive training. The general function for the prediction at step *t* is given as follows:


(5) $$fi^{\left(t\right)}=\sum_{k=1}^{t}f_{k}\left(x_{i}\right)=f_{i}^{\left(t-1\right)}+f_{t}\left(x_{i}\right)$$

where *ft (x_*i*_)* is the learner at step *t*, *f_*i*_ (t)* and *f*_*i*_ (*t*-1) are the predictions at steps *t* and *t-1*, and *x*_*i*_ is the input variable.

To avoid the overfitting problem without any influence on the model computational speed, the XGB applies the analytic expression given below to evaluate the “goodness” of the model from the original function:


(6) Obj(t)=∑k=1nl(yi-i,yi)+∑k=1tΩ(fi)

where *l* is the loss function, *n* is the number of observations, and Ω is the regularization term which is defined as follows:


(7) $$\Omega \left(f\right)=\gamma T+\frac{1}{2}\lambda {||\omega ||}^{2}$$

where ω is the vector of scores in the leaves, λ is the regularization parameter, and γ is the minimum loss needed to further partition the leaf node. More information and procedures of the computation of the XGB algorithm can be found in the study by Chen and Guestrin (1994).

#### Random Forest

The RF model was developed by Breiman (2001) and uses the “bagging” idea to ensemble a collection of decision trees with controlled variance. The RF model is commonly used for regression and prediction problems. An RF regression is a specific type of bootstrap ensembles. It deals with random binary trees that use a subset of the observations *via* bootstrapping, where a random subset of the training dataset is sampled from the raw dataset and utilized to evolve the model. The detailed computational procedure of the RF model can be found in the studies by Breiman (2001) and Ferreira and da Cunha (2020). To get the best score, an RF was trained using 200 trees, 5 max depth, and the default values of the other hyperparameters. During the tuning phase, the following sets of hyperparameters and their respective values were used: *n* estimators (number of trees) (100, 200, 300, and 500) and max depth (1, 2, 5, and 10).

#### Deep Neural Network

The DNN is a powerful DL model (Montes-Atenas et al., 2016; Achieng, 2019). It is an artificial neural network (ANN) with multiple layers between the input layers, hidden layers, and output layers to learn more complex non-linear relationships between input and output. In this study, the rectified linear unit (ReLU) was applied as an activation function which is commonly employed to establish input-output relationships and defined as follows (Xu et al., 2015; Ghimire et al., 2019):


(8) $$ReLu\left(s\right)=\left\{\begin{array}{c} x(x>0\\ 0(x\leq 0 \end{array}\right\}$$

The loss function in the DNN model is expressed as follows:


(9) $$loss=\frac{1}{2n}\sum_{i=1}^{n}{\left(T_{i}-T_{i}^{{}^{\prime }}\right)}^{2}$$

where *n* is the number of observation data *T*, and *T*′is the estimated value by the DNN model which can be defined for a three-hidden-layer DNN model with the ReLU activation function as follows:


(10) $$T^{\prime }=ReLu\varpi_{4}\left(\varpi_{3}\left(ReLu\left(\varpi_{2}\left(ReLu\right)\left(\varpi_{1}+b_{1}\right)\right)+b_{3}\right)\right)+b_{4}$$

where ω_1_, ω_2_, ω_3_, and ω_4_ are the weights in the network and b_1_, b_2_, b_3_, and b_4_ are the bias terms.

### Performance Evaluation of the Models

In this study, the mean absolute error (MAE), the root mean square error (RMSE), and the mean bias error (MBE) were used to evaluate the applied models. In addition, uncertainty with a 95% confidence level (U95) was estimated (Gueymard, 2014; Behar et al., 2015). The model deviations and the T-statistic test (Tstat) were used to evaluate the significant differences between the predicted and the observed yield (Stone, 1994; Gueymard, 2014). The performance statistics are defined as follows:


(11) $$MAE=\frac{1}{n}\sum_{i=1}^{n}\left|O_{i}-P_{i}\right|$$

(12) $$RMSE=\sqrt{\frac{1}{n}\sum {\left(P_{i}-O_{i}\right)}^{2}}$$

(13) $$MBE=\frac{1}{n}\sum_{i=1}^{n}\left(O_{i}-P_{i}\right)$$

(14) $$SI=\frac{RMSE}{O^{-}}$$

(15) $$T_{stat}=\sqrt{\frac{\left(1-n\right)MBE^{2}}{RMSE^{2}-MBE^{2}}}$$

(16) $$U_{95}=1.96\sqrt{\left(SD^{2}+RMSE^{2}\right)}$$

where Ō represents the average values of the observed yield, *O*_*i*_ and *P*_*i*_ are the observed and predicted yield, respectively, and *i* is the number of observations. *SD* is the standard deviation of the difference between the observed and estimated values. The range of the Scatter Index (SI) for the classification of the models is “excellent” if SI < 0.1, “good” if 0.1 < SI < 0.2, “fair” if 0.2 < SI < 0.3, and “poor” if SI > 0.3. Notably, the MBE and T-statistics take both negative and positive values.

In this study, the datasets were divided into 70% for training and 30% for testing. The ML models were implemented using the Python programming language library Scikit-learn 0.22.1. A virtual machine was established on Google Cloud Platform which was used for the computations. The hyperparameter tuning was performed using a grid search method for each model to get the best score as well as the best parameter sets that gave the lowest prediction errors in the testing stages (Al-Fugara et al., 2020; Fan et al., 2021). For support vector regressor (SVR), two different kernels (i.e., radial basis function and linear) were applied, as well as regularization parameter *C* from the set (1, 2, 3, 4, and 5), and maintained the default values of the remaining hyperparameters. To get the best score, an XGB was applied by using 400 trees, 10 max depths, a learning rate of 0.1, and the other hyperparameters that are the default values. The following sets of hyperparameters were applied: *n* estimators (number of trees) (100, 200, 300, 400, and 500); max depth (1, 2, 5, 10, and 12); and learning rate (0.05, 0.1, and 0.5). RF was trained using 400 trees, where 10 max depth and the default values of the other hyperparameters were used. During the hyperparameter tuning stage, the following sets of hyperparameters were assessed: number of trees (100, 200, 300, 400, and 500) and max depth (1, 2, 5, 10, and 12). For the DNN model, the neuron numbers in the four hidden layers were 256, 128, 128, and 64 neurons, respectively, and the iterations (epochs) were optimized as 500 epochs.

## Results and Discussion

### Evaluation of the Machine Learning Models

**FIGURE 3:**
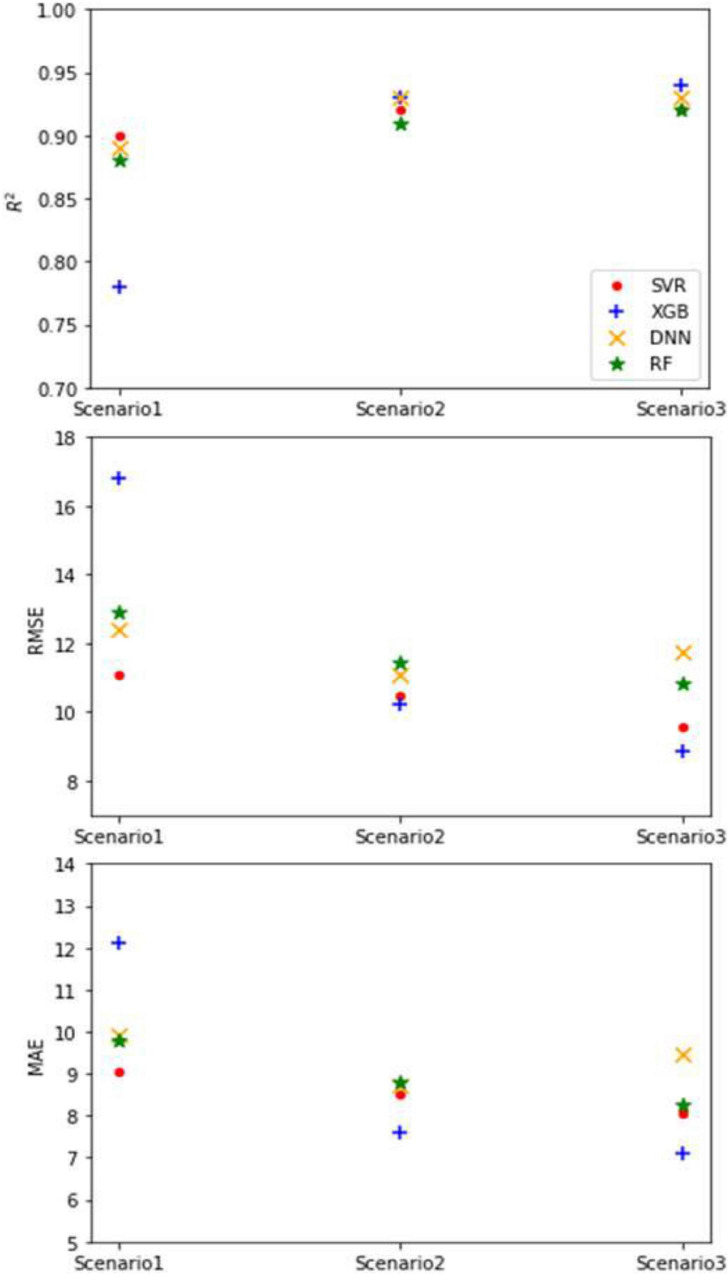
The performance statistics values for different model scenarios.

The results of the application of the ML models are shown in Figure 3. The XGB model with scenario 3 yielded the lowest RMSE value of 8.88 g followed by SVR with scenario 3 at 9.55 g, and the highest value was in XGB with scenario 1. With regard to MAE, XGB reported the lowest value with scenario 3 as 7.1 g, and the same model yielded the highest value with scenario 1 as 12.1 g. In terms of the coefficient of determination (*R*^2^), all model scenarios registered more than 0.88 except for XGB with scenario 1 which recorded a modest value of 0.78 (Figure 3).

The lowest T-statistic was recorded by SVR with scenario 2, and the highest was recorded by DNN with scenario 2. For the uncertainty, XGB with scenario 3 recorded the lowest value as 24.8, and the highest value of 46.8 was recorded by the same model but with scenario 1, following the same trend as RMSE and MAE. In terms of the MBE, the highest value was reported by the DNN model with scenario 3 as 3.95 g followed by DNN with scenario 2 as 3.8 g. All model scenarios produced SI values of <0.1, which is an indication of excellent performance by all models. This may be related to the strong correlation between the input and output variables. However, the selection of input variables is one of the most important aspects for ML models to achieve better results.

The ML models performed well at the controlled environment level. Our methodology is scalable, simple, and inexpensive for estimating lettuce fresh weight. It is observed that the prediction accuracy of the models varied and also depended on the scenario input variables. Prediction of crop yield is extremely challenging due to its dependency on multiple factors, such as crop genotype, environmental factors, management practice, and their interactions (Khaki et al., 2020). There are many studies discussing crop genotype and environmental factors, but our study is focused on the effect of plant components and water consumption on yield (fresh head weight). The DL subset of ML can be further improved by combining with crop models, adding detailed farming management data, and higher spatiotemporal input variables (Cao et al., 2021).

We predicted lettuce crop yield depending on the input variable scenarios. Scenario 1 consisted of leaf number and water consumption, scenario 2 combined leaf number, dry weight, and water consumption, and scenario 3 included all features (i.e., stem diameter, leaf number, stem length, dry weight, and water consumption). Our results are in agreement with previous studies that showed that the RF model can accurately estimate crop yields (Fukuda et al., 2013; Everingham et al., 2016). There was no overfitting during the training stage for the RF model yet it had the lowest *R*^2^ for scenarios 2 and 3 and the second lowest value after XGB for scenario 1. In contrast, the results of Jeong et al. (2016) reported that the algorithm of RF may suffer overfit to data because its algorithm consists of an ensemble of a large number of decision trees that may not be fully described mechanistically. Also, RF may cause a loss of accuracy when extreme ends are expected or responses are outside the limits of the training data (Jeong et al., 2016).

### Model Comparison

**FIGURE 4:**
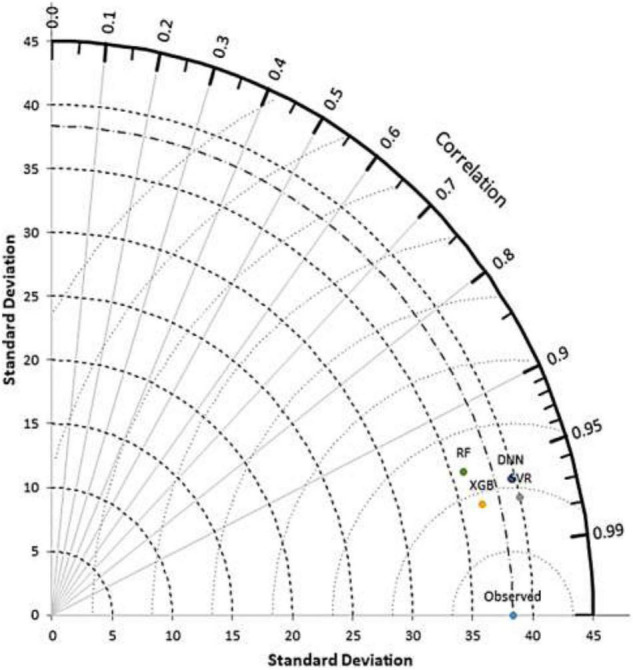
Taylor diagram displaying a statistical comparison of the applied models used for predicting fresh head weight (yield).

As shown in Figure 3, the XGB model reported the lowest RMSE and MAE values of 2.69 and 2.2%, respectively, and also the highest *R*^2^ value (0.94) for scenario 3. According to the SI statistics, the SVR model with scenario 3 had excellent performance (Li et al., 2013). The second model was XGB as judged by the RMSE (2.89%) and MAE (2.4%) performance statistics. Figure 4 presents a Taylor diagram that shows how much the observations are matched by the predictions and the degree of compliance by the model (Taylor, 2001; Maroufpoor et al., 2019). It is clear that the best models were SVR with scenario 3 and DNN with scenario 2. However, SVR with scenario 3 (i.e., leaf number, water consumption, dry weight, stem length, and stem diameter) is superior, and DNN with scenario 2 (i.e., leaf number, water consumption, and dry weight) is equally good. It needs to be mentioned that DNN with scenario 2 has less input features than SVR with scenario 3, making DNN with scenario 2 the preferred model. Nevertheless, all four models that were applied have a high correlation coefficient in excess of 0.95, and the SD was close to the observed values.

**FIGURE 5:**
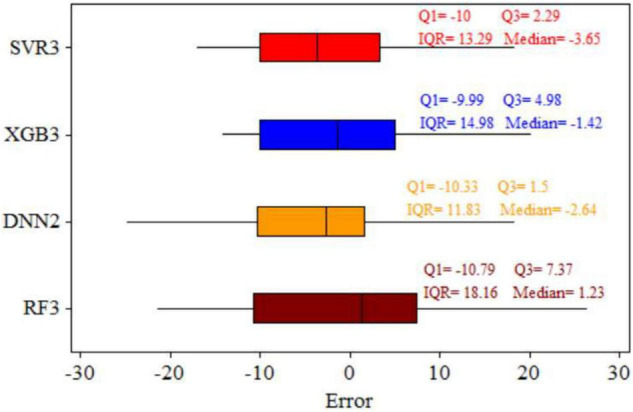
Boxplots showing the distribution of the estimation errors in the test stage for support vector regressor (SVR), extreme gradient boosting (XGB), deep neural network (DNN), and random forest (RF) models. Q25, lower quartile of errors; Q75, upper quartile of errors; IQR, interquartile range for each model.

A boxplot to compare the models based on the residuals (estimation error) is shown in Figure 5. Positive and negative estimation errors show under- and overestimations, respectively. The DNN with scenario 2 model appears to be the best model having the lowest error in comparison with the others. It has a lower quartile (Q1) value of –10.33, while XGB has a value of –9.99, and SVR, a value of –10. Third quartile (Q3) error analysis is better than Q1 because it contains 75% of the error. It is reported that the DNN with scenario 2 model has a difference of ΔQ3 = 3.48 compared with XGB with scenario 3 which has ΔQ3 = 0.79 compared with SVR3. Moreover, the smaller interquartile range (IQR = Q3–Q1) by DNNs compared with the other three models clearly show that its distribution of error is much better than the others (Figure 5), and it is therefore preferred.

As mentioned earlier, the highest *R*^2^ and the lowest RMSE were recorded by XGB (0.94 and 8.88, respectively) with scenario 3, followed by DNN with scenario 2 (0.93 and 11.11, respectively). Also, XGB with scenario 3 had the lowest MAE followed by XGB with scenario 2. These results do not agree with Fan et al. (2021) who reported that the best model results were given by DNN models (*R*^2^ = 0.816–0.954), slightly outperforming SVR models (*R*^2^ = 0.731–0.948) during the testing stage, followed by XGB models (*R*^2^ = 0.739–0.929) under the four-input combination, but their research was about summer maize in Northwest China. The DNN model had a high prediction performance of yield which is similar to those reported by Khaki and Wang (2019), where RMSE for the validation dataset was around 11% of their respective values. The accuracy for the prediction of the crop yield was slightly higher than that reported by Khaki and Wang (2019) because they used average yield. In Table 3, the SI values are lower than 0.1 for all model scenarios, meaning the accuracy of the models can be characterized as “excellent” (Li et al., 2013; Maroufpoor et al., 2019).

**TABLE 3:** The performance statistics of support vector regressor (SVR), extreme gradient boosting (XGB), deep neural network (DNN), and random forest (RF) models for lettuce.

Model	Scenario	SI	T	U95	MBE
SVR	1	0.035	0.647	31.90	1.59
	2	0.032	0.015	29.35	0.034
	3	0.029	1.600	26.10	3.10
XGB	1	0.051	0.780	46.80	2.84
	2	0.031	0.110	28.70	–0.25
	3	0.027	0.540	24.80	1.04
DNN	1	0.037	0.630	34.50	1.70
	2	0.033	1.650	30.30	3.80
	3	0.035	1.630	31.90	3.95
RF	1	0.039	0.160	36.2	–0.45
	2	0.035	0.135	32.1	–0.34
	3	0.033	0.087	30.3	–0.21

*SI, Scatter Index; Tstat, T-statistic test; U95, Uncertainty with a 95% confidence level; MBE, mean bias error.*

## Conclusion

This study presented ML approaches for the prediction of lettuce crop yield cultivated in three different hydroponic systems which interacted with three different kinds of magnetic water. Three samples were collected from each system 50 days after transplanting, at the same time, for all systems for 2 years. The datasets were divided into 70% for the training of the four ML models (i.e., RF, XGB, SVR, and DNNs) used to predict lettuce crop yield based on the three scenarios of input plant and water features, and 30% of the remaining data were used for testing the models.

The lowest RMSE was recorded in XGB with scenario 3 followed by SVR with scenario 3, and the highest, by RF with scenario 1. The *R*^2^ was more than 0.77 for all applied model scenarios. Based on the SI, all models performed excellently, especially XGB with scenario 3 and SVR with scenario 3. Based on all performance statistics, the two best models were SVR with scenario 3 and DNN with scenario 2. However, the latter model scenario is preferred because it requires fewer input variables.

The methods developed in this study can be further improved by combining the input variables with climate variables, farming management data, and higher resolution spatiotemporal input variables for the successful prediction of crop yield on a large scale. The ML models could be a rapid tool for predicting crop yield and disaster evaluation over a large area.

## Code Availability

Codes and datasets generated and/or analyzed during this study are available from the corresponding author on reasonable request.

## Data Availability Statement

The original contributions presented in the study are included in the article/supplementary material, further inquiries can be directed to the corresponding authors.

## Ethics Statement

This study was approved by the Agricultural Engineering Research Institute, Agricultural Research Center, and the authors certify that this study was performed in accordance with the ethical standards as laid down in the 1964 Declaration of Helsinki and its later amendments or comparable ethical standards. The participants provided their written informed consent to participate in this study.

## Author Contributions

WE-S collected and analyzed the research data. AM designed and applied the ML models of the research. WE-S, AM, and MA wrote the original manuscript and provided suggestions on data analysis. NA-A, SS, and YG-A edited and provided suggestions to improve the content and structure of the manuscript. All authors read and edited the final manuscript before submission.

## Conflict of Interest

The authors declare that the research was conducted in the absence of any commercial or financial relationships that could be construed as a potential conflict of interest.

## Publisher’s Note

All claims expressed in this article are solely those of the authors and do not necessarily represent those of their affiliated organizations, or those of the publisher, the editors and the reviewers. Any product that may be evaluated in this article, or claim that may be made by its manufacturer, is not guaranteed or endorsed by the publisher.
